# Comparing Blanket vs. Selective Dry Cow Treatment Approaches for Elimination and Prevention of Intramammary Infections During the Dry Period: A Systematic Review and Meta-Analysis

**DOI:** 10.3389/fvets.2021.688450

**Published:** 2021-06-15

**Authors:** Fidèle Kabera, Jean-Philippe Roy, Mohamed Afifi, Sandra Godden, Henrik Stryhn, Javier Sanchez, Simon Dufour

**Affiliations:** ^1^Département de Pathologie et Microbiologie, Faculté de Médecine Vétérinaire, Université de Montréal, Saint-Hyacinthe, QC, Canada; ^2^Mastitis Network, Saint-Hyacinthe, QC, Canada; ^3^Département de Sciences Cliniques, Faculté de Médecine Vétérinaire, Université de Montréal, Saint-Hyacinthe, QC, Canada; ^4^Department of Health Management, Atlantic Veterinary College, University of Prince Edward Island, Charlottetown, PE, Canada; ^5^Department of Animal Wealth Development, Biostatistics, Faculty of Veterinary Medicine, Zagazig University, Zagazig, Egypt; ^6^Department of Veterinary Population Medicine, College of Veterinary Medicine, University of Minnesota, St. Paul, MN, United States

**Keywords:** dairy cows, dry period, selective antimicrobial treatment, intramammary infection, antimicrobial use

## Abstract

A systematic review and a series of meta-analyses were conducted to investigate the efficacy of selective dry cow antimicrobial treatment (SDCT) (in which only infected quarters/cows were treated with an antimicrobial) compared with blanket dry cow treatment (BDCT) (all quarters/all cows received an antimicrobial, regardless of their infection status). A full detailed protocol was published before initiating this review. Studies reporting on the (1) proportion of untreated quarters or cows when using SDCT, (2) intramammary infection (IMI) incidence risk over the dry period, (3) IMI elimination risk, (4) post-calving IMI prevalence, (5) early lactation clinical mastitis incidence, or (6) subsequent lactation milk yield and somatic cell counts were considered eligible. Thirteen articles representing 12 controlled trials, whether randomized or not, were available for analyses. SDCT reduced the use of antimicrobials at dry off by 66% (95% CI: 49–80). There was no difference in the elimination of existing IMI at dry off, between SDCT and BDCT. Meta-regression showed that the risk of IMI incidence during the dry period, IMI risk at calving, early lactation clinical mastitis risk, and early lactation milk yield and somatic cell counts did not differ between SDCT and BDCT as long as an internal teat sealant (65% bismuth subnitrate) was administered to untreated healthy quarters/cows at dry off. For trials not using internal teat sealants, SDCT resulted in higher risk than BDCT of acquiring a new IMI during the dry period and of harboring an IMI at calving. Lines of evidence strongly support that SDCT would reduce the use of antimicrobials at dry off, without any detrimental effect on udder health or milk production during the 1st months of the subsequent lactation, if, and only if, internal teat sealants are used for healthy, untreated quarters/cows.

## Introduction

Blanket dry cow therapy (BDCT), where all quarters of all cows are treated with a long-acting antimicrobial at dry off, was introduced many years ago (1) and is widely used by dairy farmers. This practice permits to increase the elimination of existing intramammary infections (IMI) at dry off and prevent the occurrence of new IMI during the dry period. In fact, persistent and new IMI during the dry period can result in the development of clinical mastitis (CM) early in the next lactation (2–4).

However, with changes in mastitis epidemiology and increasing public health concerns regarding the use of antimicrobials in food-producing animals, selective dry cow therapy (SDCT) is a potential alternative to BDCT to reduce antimicrobial usage in dairies (5–7). With the SDCT approach, antimicrobial treatment is reserved for cows or quarters suspected of having an IMI, while uninfected cows and quarters usually do not receive an antimicrobial treatment. In addition, internal teat sealants (ITS) have been shown to be a very effective non-antimicrobial alternative to prevent new IMI during the dry period (8–10). A teat sealant could be used to protect untreated cows or quarters when a selective antimicrobial treatment approach at dry off is applied. Thus, SDCT could prevent the use of antimicrobials for a prophylactic purpose and that it could possibly be without detrimental changes to udder health parameters (11).

A systematic review comparing blanket and selective dry cow therapy and describing the various advantages and potential negative impacts would be of great importance for decision-makers to engage in an effective and judicious use of antimicrobials at dry off. Recently, a systematic review (12) reported on the impact of selective vs. blanket dry cow therapy, but on only one outcome, prevalence of IMI at calving. In this latter study, reduction in the use of antimicrobials at dry off (the main reason for choosing SDCT) was not investigated, nor was the risk of CM, milk yield, or somatic cell counts (SCC) in the early next lactation. These outcomes are all very important for choosing the best dry cow treatment protocol. Moreover, IMI dynamics during the dry period (i.e., acquisition and elimination of IMI during the dry period) was not investigated in the study of Winder et al. (12). Nevertheless, studying IMI dynamics can provide better insights on the underlying biological processes, compared with studying prevalence at a single point in time (e.g., at calving).

### Objective

The objective of the current study was to investigate the efficacy of SDCT, compared with the treatment of all quarters of all cows, for (1) reducing the use of antimicrobials at dry off, (2) preventing IMI incidence during the dry period, (3) eliminating existing IMI at dry off, (4) reducing the prevalence of IMI at calving, and (5) preventing early lactation CM. Another objective was also to investigate whether milk yield and SCC in the early lactation would be affected. Our hypothesis was that SDCT protocols could be implemented without negative health or production effects and would result in a substantially lower usage of antimicrobials at dry off.

The population, intervention, comparator, outcome (PICO) questions answered by the current study were formulated as: in dairy cows (i.e., the population), is SDCT (i.e., the intervention) as efficient as BDCT (i.e., the comparator), (1) in preventing new IMI during the dry period, (2) in eliminating existing IMI at dry off, (3) in reducing IMI risk at calving, and (4) in preventing early lactation CM; and (5) what are the impacts of dry cow treatment approach on milk yield and SCC in the early lactation (i.e., the outcomes)?

## Methods

This current systematic review was reported using the Preferred Reporting Items for Systematic Reviews and Meta-Analyses (PRISMA) statement guidelines (13). The detailed protocol for this review was published elsewhere prior to initiating the review (14). The complete protocol targeted three independent objectives: (1) choice of antimicrobial at drying off, (2) comparison of blanket vs. selective dry cow treatment, and (3) complementing an antimicrobial treatment with a teat sealant. However, the current manuscript reports only on the comparison of blanket and selective dry cow treatments. The other two objectives will be addressed in two future independent manuscripts.

The complete search strategy described in the published protocol was initially conducted on May 1st, 2018, and updated on June 16th, 2020, prior to finalizing the analyses and manuscript. The search strategies were all conducted on the same day for the three electronic sources of information (Medline, CAB Abstracts, and Web of Science) and for conference proceedings from the National Mastitis Council and the American Association of Bovine Practitioners. Modifications and precisions to the published protocol with their justifications are described in the following sections.

### Modifications and/or Precisions to the Published Protocol

#### Eligibility Criteria

In the published protocol, we planned to include studies where the post-calving IMI status was determined within 14 days in milk (DIM), to ensure that the new IMI or elimination most likely occurred during the dry period (vs. in the early lactation). However, in some articles, cows were sampled twice after calving (for instance, 3–4 DIM and 5–18 DIM), and a parallel interpretation of the two milk samples was used to define IMI status. Hence, some studies relied on testing within an interval that extended slightly beyond 14 DIM but was mostly within 0–14 DIM. We decided to retain these studies (5, 6, 15). In the published protocol, we indicated CM incidence during the first 0–4 months after calving as a studied outcome. More precisely, we did use the CM data from studies with a shorter follow-up period and otherwise extracted the data up to a maximum of 4 months in milk.

#### Risk of Bias in Individual Studies

As it was planned in the protocol, we proposed to record different domains of risk of bias (ROB) by outcome's type. In fact, the ROB 2.0 makes it clear that the assessment is typically specific to a particular result, and consequently, the assessments of ROB need to be outcome-specific (16). However, all measured outcomes yielded the same evaluation within a given trial. Hence, for simplicity, we only reported the risk for a domain for all outcomes of a trial at once. As all the included studies were controlled trials (whether randomized or not), only the Cochrane Collaboration's tool for assessing ROB was used for assessing ROB in selected studies (Cochrane Handbook for Systematic Reviews of Interventions, version 5.1.0).

#### Summary Measures

Daily mean milk production (kg/day) or mean ln SCC during the first 4 months was extracted directly from included trials or obtained from personal communications with the authors. Thus, raw mean difference (MD) was used as the effect size, for those two outcomes.

#### Data Synthesis and Meta-Analysis

Meta-analyses were conducted in R version 4.0.0 [R Foundation for Statistical Computing Platform: ×86_64-w64-mingw32/×64 (64-bit)] using RStudio version 1.2.1335 (RStudio Inc., Boston, MA, USA) using the “meta” package version: 4.12-0 (2020-05-04). Studies were weighed using the inverse variance method based on the logit transformation. A random effects approach was used, as it was described in the published protocol (14) and the between-study variability was estimated using the method of restricted maximum likelihood (REML) (17) and the Knapp–Hartung adjustment for random effects model (18). Heterogeneity was assessed by the *I*^2^ statistic. Effects of trial level characteristics were tested using a meta-regression model with one covariate and only if at least three trials were included in each category of the covariate.

#### Confidence in Cumulative Estimates

The Grading of Recommendations Assessment, Development, and Evaluation (GRADE) approach involves rating, for each comparison made, the confidence in effect estimate based on an assessment of eight domains: number of trials, ROB, inconsistency, indirectness, imprecision, publication bias, number of individuals (in our case quarters or cows) followed, and a summary measure of association with its 95% CI. Then, an overall assessment is made regarding the level of confidence in the summary effect estimate observed. For rating the different domains of the GRADE, in the current review, we used the guidelines suggested by Dufour et al. (8).

Briefly, for the ROB domain, a trial was rated at low ROB, when at least four out of seven evaluated domains for an individual trial were rated at low risk with a maximum of one domain evaluated at high risk. When at least four domains were rated at low risk but with two domains evaluated at high risk, the trial was rated at moderate ROB. In other cases, the trial was rated at high ROB. For the inconsistency domain, we visually appraised, using forest plot, whether a uni-, bi-, or multimodal distribution of point estimates was observed across trials and rated these, respectively, as no serious, serious, and very serious limitations. Regarding the indirectness domain, we computed independently the proportion of trials for which the investigated population, intervention, and outcome matched those of interest, and an equal weight was given to these three subdomains. Comparisons with a score ≥66%, between 65 and 33%, and of ≤ 33% for that domain were then rated as no serious, serious, and very serious limitations, respectively.

For the imprecision domain, the difference between the natural logarithm of the higher and lower bounds of the summary relative risk (RR) was computed. Comparisons with confidence interval bounds, differences ≤ 1.1 on the logarithmic scale (equivalent to an RR interval of 1.0–3.0 points), between 1.1 and 1.6 (equivalent to an RR interval of 3.0–5.0 points), and ≥1.6 (equivalent to an RR interval of ≥5.0 points) were rated, respectively, as no serious, serious, and very serious limitations. For the imprecision domain for milk yield and SCC, in addition to the examination of upper and lower limits of the 95% confidence intervals, we considered the calculation of an optimal information size (19). When the optimal information size criterion was not met, the precision was rated as serious limitations. When the optimal information size criterion was met and the 95% CI length <2 (i.e., a mean difference of −1.00 to +1.00) for milk yield and ln SCC, we rated precision as no serious limitations. When the optimal information size criterion was met, and the 95% CI length ≥2 and ≤ 4 for milk yield and ln SCC, the precision was rated as serious limitations. When the optimal information size criterion was met, and the 95% CI length ≥4 for milk yield and ln SCC, the precision was rated as very serious limitations.

Finally, for the publication bias domains, we considered whether the number of trials allowed us to fully appraise funnel plot asymmetry. We also considered whether the outcomes studied would be associated with any commercial advantages.

## Results

### Study Selection

**Figure 1:**
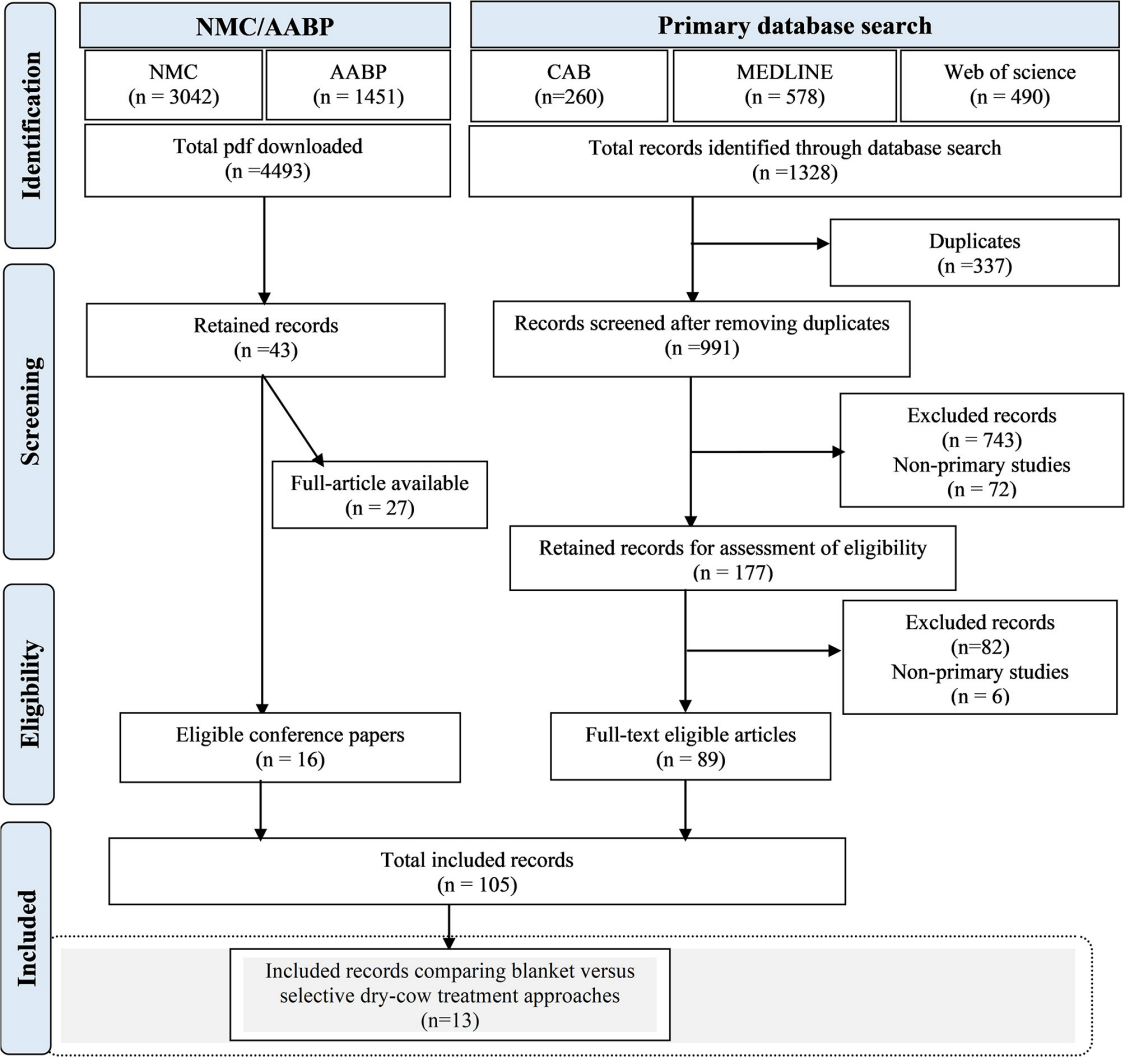
Result of the different steps for searching and identifying relevant records for the systematic review and meta-analysis on antimicrobial-based dry cow therapy approaches. The search was conducted to answer three research objectives: (1) choice of antimicrobial at drying off; (2) comparison of blanket vs. selective dry cow treatment; and (3) complementing an antimicrobial treatment with a teat sealant. The gray box indicates results specific for objective (2), comparison of blanket vs. selective dry cow treatment, and the other two objectives will be presented in subsequent independent articles. Screening of references cited by the included articles was also conducted but did not lead to the addition of eligible articles specific to the comparison of selective and blanket dry cow therapies. This latter part of the search strategy will be presented for the other two objectives in the subsequent associated articles. NMC, National Mastitis Council; AABP, American Association of Bovine Practitioners.

Results of the different steps for searching and assessing eligibility of studies are presented in Figure 1. After removing duplicates and exclusion due to language restriction, a total of 991 records were identified from three databases: CAB Abstracts, Web of Science, and Medline. Of the 991 records, after reviewing the content of the abstracts and full texts, only 89 records met the inclusion criteria for at least one of the PICO questions on antimicrobial-based dry cow therapy approaches.

In addition, 43 records were identified through the search of the National Mastitis Council (NMC) and American Association of Bovine Practitioners (AABP) conference proceedings. Finally, after excluding proceeding papers for which an equivalent full article was available (*n* = 27), 105 records combining 89 full articles and 16 conference papers were included.

The references cited in these 105 retained records and 78 non-primary studies were screened for any additional relevant study which was not initially retrieved through the databases or conference proceedings search, but no additional eligible records were identified from this process for the comparison of SDCT and BDCT.

Of the 105 records retained, 13 articles representing 12 trials reported on the comparison of SDCT and BDCT and, therefore, were included in this part of the systematic review. Other retained records will be discussed in two other manuscripts reporting on the choice of antimicrobial at drying off or on complementing an antimicrobial treatment with an ITS.

### Included Studies

Characteristics of the 13 included articles representing 12 trials are described in Table 1. Those 12 trials include six trials reported in six articles (7, 15, 20, 23, 24, 26), two trials where each trial was reported in two articles for different outcomes (5, 21, 22, 25), two trials reported in two articles where each article reported on both trials (27, 28), and two trials reported in one article (6). Furthermore, the description of the SDCT group and of the reported outcomes are summarized in Table 2. Finally, the follow-up period after calving and the definitions of IMI at dry off and calving, of new IMI, and of elimination of IMI during the dry period used in each study are provided as (Supplementary Table 1).

**Table 1:** Characteristics of 13 articles representing 12 trials included in the systematic review comparing selective dry cow therapy and blanket dry cow therapy for curing and preventing intramammary infections.

**References**	**Country**	**Study design**	**#^**a**^ herds**	**# cows**	**# quarters**	**Inclusion criteria**
Ward and Schultz (15)	USA	CT	4	402	1,600	No criteria
Roguinsky and Serieys (20)	France	CT	1	40	159	NR
Rindsig et al. (7)	USA	CT	1	232	928	NR
Browning et al. (21, 22)	Australia	CT	12	1,044	4,176	BTSCC 100,000–400,000 cells/ml; cow's expected dry period ≥2 months; and <4 infected quarters at dry off
Williamson et al. (23)	New Zealand	CT	4	371	NR	NR
Hassan et al. (24)	Australia	CT	3	150	600	NR
Cameron et al. (5, 25)	Canada	RCT	16	603	2,287	BTSCC < 250,000 cells/ml; cow's SCC < 200,000 cells/ml on the last three DHI tests; no CM on the same period; cow's expected dry period 30–90 days; cow had no antimicrobial treatment in the last 14 days; all quarters of the cow had CMT < 2 on the day prior to drying off.
Patel et al. (26)	USA	RCT	1	56	224	Four functional quarters; no antibiotic or anti-inflammatory medication during the 14-day period prior to dry off; clinically healthy; no signs of CM at enrollment or on the day of dry off; expected dry period 30–90 days
Rowe et al. (27, 28)	USA	RCT	7	1,243	5,100	Herd size sufficient to dry off ≥ 15 cows per week; BTSCC < 250,000 cells/ml; record CM, culling, and death events; cow's expected dry period 30–90 days; no antibiotic or anti-inflammatory treatment within 14 days; no CM; no lameness (>3/5) or poor body condition (<2/5)
Kabera et al. (6)	Canada	RCT	9	569	2,142	BTSCC < 250,000 cells/ml; no CM or antimicrobial treatment during 14 days prior to dry off; and cow's expected dry period 35–75 days

*CT, controlled trial (no randomization reported); NR, not reported; BTSCC, herd mean 12-month bulk-tank somatic cell count; RCT, randomized controlled trial; SCC, somatic cell counts; DHI, dairy herd improvement; CM, clinical mastitis; CMT, California Mastitis Test*.

a *Number of units analyzed*.

**Table 2:** Treatment regimens and outcomes studied in 13 articles representing 12 trials included in a systematic review comparing selective dry cow therapy (SDCT) and blanket dry cow therapy (BDCT).

**References**	**SDCT description**						**Outcomes measured**			
	**Method for identifying units to treat**	**Level^**a**^**	**Threshold for treatment**	**Tx^**b**^ if +**	**Tx^**c**^ if –**	**% with no ATB^**d**^**	**New IMI^**e**^**	**Elimination of IMI^**f**^**	**IMI^**g**^**	**Others in next lactation**
Ward and Schultz (15)	CM^h^	Q	≥1 CM in last month	Neomycin sulfate	No Tx	96.1	Yes	Yes	Yes	CM
Roguinsky and Serieys (20)	CMT	C	≥1 quarter with CMT ≥3 in last month	Cloxacillin or penicillin and streptomycin (half of the cows received each treatment)	No Tx	68.2	Yes	Yes	Yes	None
Rindsig et al. (7)	SCC, CMT, and CM	C	Cow SCC > 500,000 cells/ml or CMT ≥ 2 in any quarter or ≥1 CM	Penicillin and streptomycin	No Tx	42.9	Yes	Yes	Yes	SCC
Browning et al. (21, 22)	Lab-based milk culture	Q	NR	Benzathine cloxacillin	No Tx	67.5	Yes	Yes	Yes	CM
Williamson et al. (23)	Lab-based milk culture	Q	NR	Cephalonium	No Tx	NR	Yes	No	No	CM
Hassan et al. (24)^*^	N-acetyl-beta-D-glucosaminidase	Q	High NAGase on a sample taken 24 h before dry off	Benzathine cloxacillin	No Tx	81.1	No	No	Yes	CM
Cameron et al. (5, 25)	Aerobic count Petrifilm	C	≥50 CFU/ml in composite milk	Ceftiofur hydrochloride and ITS	ITS	45.6	Yes	Yes	Yes	MY, CM, SCC
Patel et al. (26)	Minnesota Easy culture system	Q	≥100 CFU/ml in quarter milk	Ceftiofur hydrochloride + ITS	ITS	48.1	Yes	Yes	Yes	CM
Kabera et al. (6)	Aerobic count Petrifilm	Q	≥50 CFU/ml in quarter milk	Penicillin G procaine and novobiocin	ITS	57.4	Yes	Yes	No	MY, CM, SCC
	Aerobic count Petrifilm	Q	≥50 CFU/ml in quarter milk	Penicillin G procaine and novobiocin + ITS	ITS	58.6	Yes	Yes	No	MY, CM, SCC
Rowe et al. (27, 28)	Minnesota Easy® 4Cast® plate	Q	≥100 CFU/ml in quarter milk	Ceftiofur hydrochloride + ITS	ITS	55.5	Yes	Yes	Yes	MY, CM, SCC
	Algorithm (SCC + CM)	C	≥2 CM during lactation or any DHIA test with SCC > 200,000 cells/ml during lactation	Ceftiofur hydrochloride + ITS	ITS	55.2	Yes	Yes	Yes	MY, CM, SCC

*CMT, California mastitis test; SCC, somatic cell counts; NR, not reported; CFU/ml, colony forming units per milliliter; ITS, internal teat sealant (65% bismuth subnitrate); MY, milk yield; DHIA, Dairy Herd Improvement Association*.

a *Selection for treatment applied at the cow (C) or quarter level (Q)*.

b *Treatment for infected cow/quarter*.

c *Treatment for uninfected cow/quarter*.

d *Percentage of antimicrobial use reduction*.

e *New intramammary infections during the dry period*.

f *Elimination of intramammary infections during dry period*.

g *Prevalence of intramammary infections at calving*.

h *Clinical mastitis history in current lactation*.

* *This study had both a positive and a negative control group*.

Briefly, six included trials were randomized controlled trials and six did not clearly report a randomization process and were, therefore, considered simply as controlled trials. Seven trials reported using herd and/or cow level recruitment criteria, one trial did not set criteria to recruit cows and/or quarters, and four trials did not report on selection criteria. One trial set a selection criteria at the herd level only, while the other six trials set them both at cow and herd levels. The most common herd-level selection criteria was to have a bulk milk SCC below a predetermined threshold (ranging from 250,000 to 400,000 cells/ml). For cow-level criteria, having a standard expected dry period length was often used, as well as no recent treatment prior to dry off, and no CM at dry off. Among the six trials where breed was reported, three were conducted in crossbred and purebred (Holstein and Holstein–Jersey or Friesian and Friesian–Jersey), while the other three were conducted solely in purebred cows (Holstein/Friesian). Of the 12 trials, the selection approach was based at the quarter level for eight trials and at the cow level for the other four trials.

In all trials, measures of new IMI, of IMI elimination, and of prevalence of IMI were based on bacteriological culture of milk samples collected before drying off and after calving. Predry off samples were taken within 1 month before dry off, and days in milk at post-calving sampling ranged from 0 to 4 weeks. Of the 12 included trials, IMI incidence during the dry period was the most often reported outcome (*n* = 11), followed by elimination of IMI during the dry period (*n* = 10) and prevalence of IMI at calving (*n* = 9). Clinical mastitis in the subsequent lactation was reported in 10 trials. However, two of the 10 trials reporting on CM in the subsequent lactation were excluded from the meta-analysis, as the follow-up period was more than 4 months and it was not possible to have data specifically for the 0–120 DIM period.

Four trials reported daily mean milk production during the first 120 DIM of the subsequent lactation (6, 28), and one trial reported daily mean milk production during the first 180 DIM (25). Six trials reported on SCC during the subsequent lactation. One trial reported on an arithmetic mean scale for the 1st week and between 28 and 56 days after calving (7). One trial reported test-day ln SCC 0–180 DIM (25), two on mean milk somatic cell score for 0–120 DIM (6), and two on SCC geometric mean for 0–120 DIM (28). After contacting the authors, data could be obtained on the same scale (mean ln SCC) for five trials. Moreover, for the trial reporting on a period of 0–180 DIM, we were able to obtain data specifically for the 0–120 DIM period. This latter trial was, therefore, included in the meta-analysis comparing mean milk yield and ln SCC between SDCT and BDCT.

### Risk of Bias Within Studies

**Figure 2:**
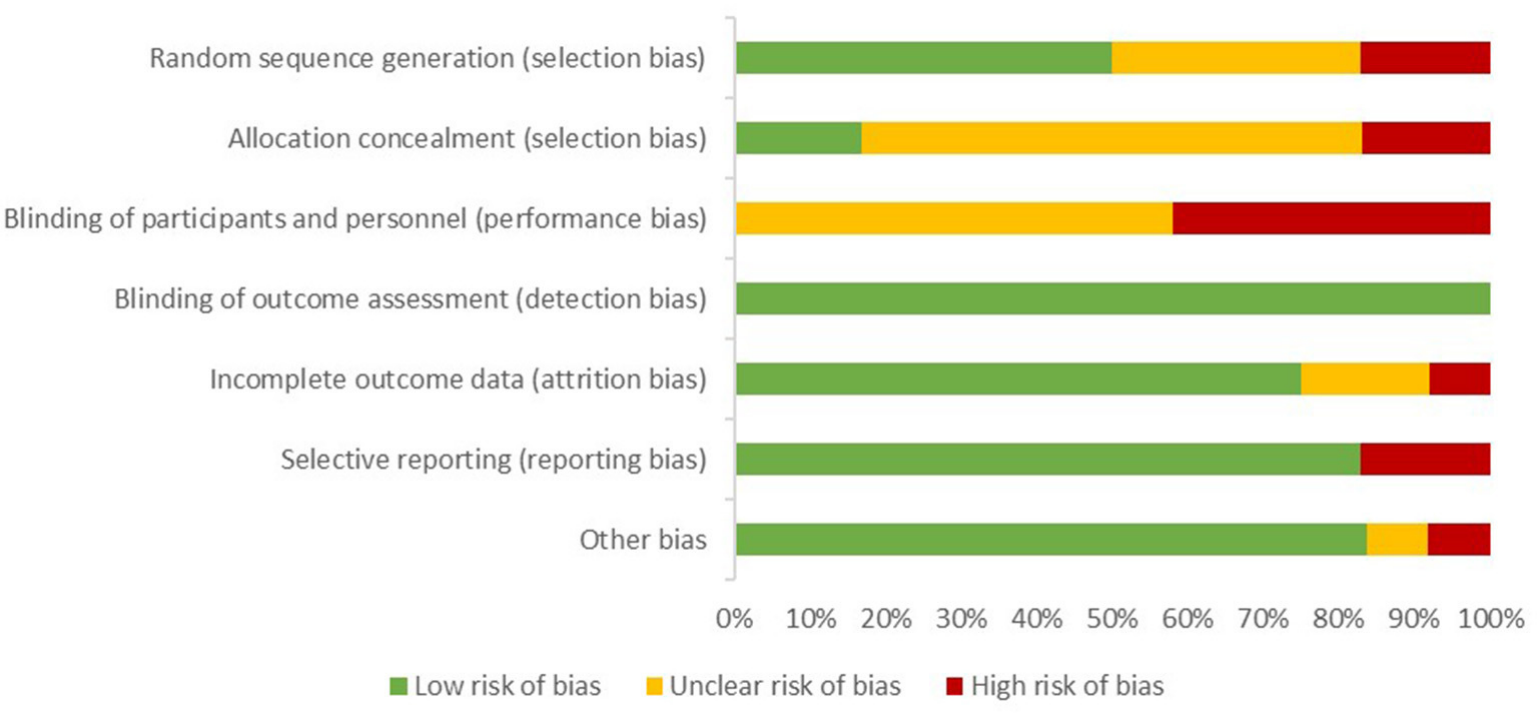
Proportion of studies with a given risk of bias among 12 trials included in a systematic review comparing selective dry cow therapy and blanket dry cow therapy.

The ROB for each individual study is reported as Supplementary Figure 1. A summary of the ROB assessment for the 12 trials included in the meta-analysis is presented in Figure 2. All trials had at least one potential source of bias rated as high or unclear. The ROB was evaluated for 13 articles reporting on 12 trials and the components with the smallest proportion of low risk trials were blinding of participants and personnel (0/12), then allocation concealment (2/12), and finally, random sequence generation (6/12). The method used to generate a random sequence was described for only six trials (5, 6, 25–28). Two trials had cows allocated alternately to two treatment groups and were consequently assessed as “high risk” (7, 15), and four other trials did not report on the randomization process in sufficient details for assessing potential bias (20–24). The allocation concealment was not described at all in eight trials. Consequently, they were classified as having an unclear risk regarding potential source of bias. It was appraised as “low risk” in two trials (6) and as “high risk” in two other trials where cows were allocated alternately (7, 15). Similarly, blinding of participants and personnel was not mentioned in seven trials. This latter component was evaluated at high risk in five other trials, as producers were not blinded to the treatment, and thus, we could not exclude an influence on the management of cows in different treatment groups. Bias due to blinding of outcome assessment (detection bias) was considered “low risk” in all trials relying mainly on laboratory analyses, which was considered to be an objective measurement.

### Meta-Analyses Comparing Selective and Blanket Dry Cow Therapies

A total of 12 trials reported on the effect of SDCT on IMI during the dry period and on udder health and milk production in the subsequent lactation, in comparison with BDCT. In addition to a positive control group (BDCT), one trial (24) included a second control group where cows did not receive any therapy at dry off. Data from this control group were not extracted, as our focus was the comparison between SDCT and BDCT.

The most important study characteristics suspected as potential sources of heterogeneity and tested in meta-regression were (1) method used to identify infected cows/quarters at dry off (milk culture vs. combination of SCC and/or history of clinical mastitis and/or California Mastitis Test and/or N-acetyl-beta-D-glucosaminidase), (2) whether the selective treatment was applied at the cow or quarter level, and (3) whether an ITS was applied for healthy cows/quarters. Meta-regression by the preceding variables was attempted if at least three trials were included in each category. For all the meta-analyses conducted, results by category of the covariate were presented rather than a general summary measure, whenever a variable tested in a meta-regression yielded a *p* < 0.05.

#### Reduction of Antimicrobial Use at Dry Off

Eleven trials reported on the reduction of antimicrobial use; however, only 10 of them could be used to summarize reduction of usage of antimicrobial at dry off. In fact, one of the trials (5, 25) reported on the reduction in the use of antimicrobials in cows preselected [individual SCC <200,000 cells/ml and no CM on the last three dairy herd improvement (DHI) tests; (Table 1) before the randomization into selective and blanket treatment groups. Thus, it was not comparable with other trials, regarding the reduction of antimicrobial use.

**Figure 3:**
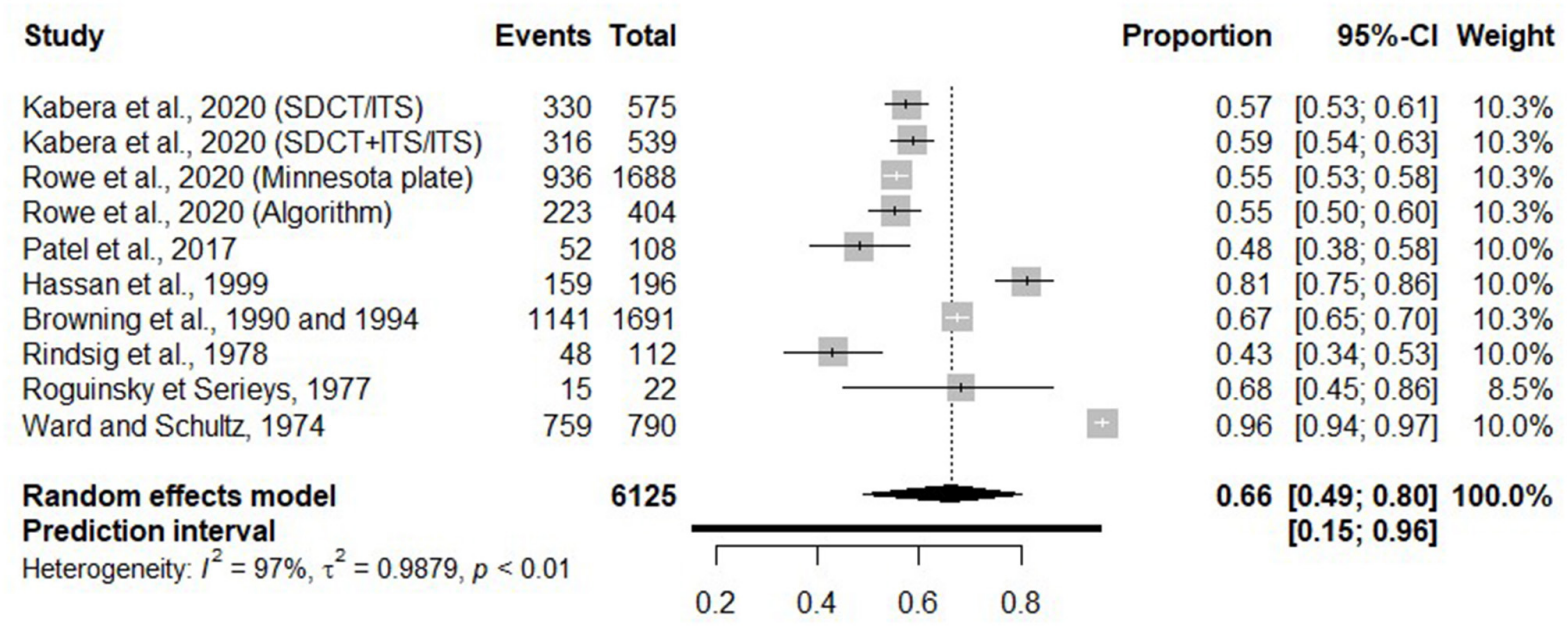
Forest plots showing the proportion of antimicrobial use reduction.

Three trial characteristics [diagnostic test used to identify infected cows/quarters at dry off; whether the selective antimicrobial treatment was applied at cow or quarter level and whether an ITS was applied for untreated (healthy) cows/quarters or not] were tested. None of them could explain the observed heterogeneity (*I*^2^ = 97%). Figure 3 presents the proportion of antimicrobial use reduction for each trial and a summary measure.

#### Effects of Selective Dry Cow Therapy on IMI Incidence Over the Dry Period

**Figure 4:**
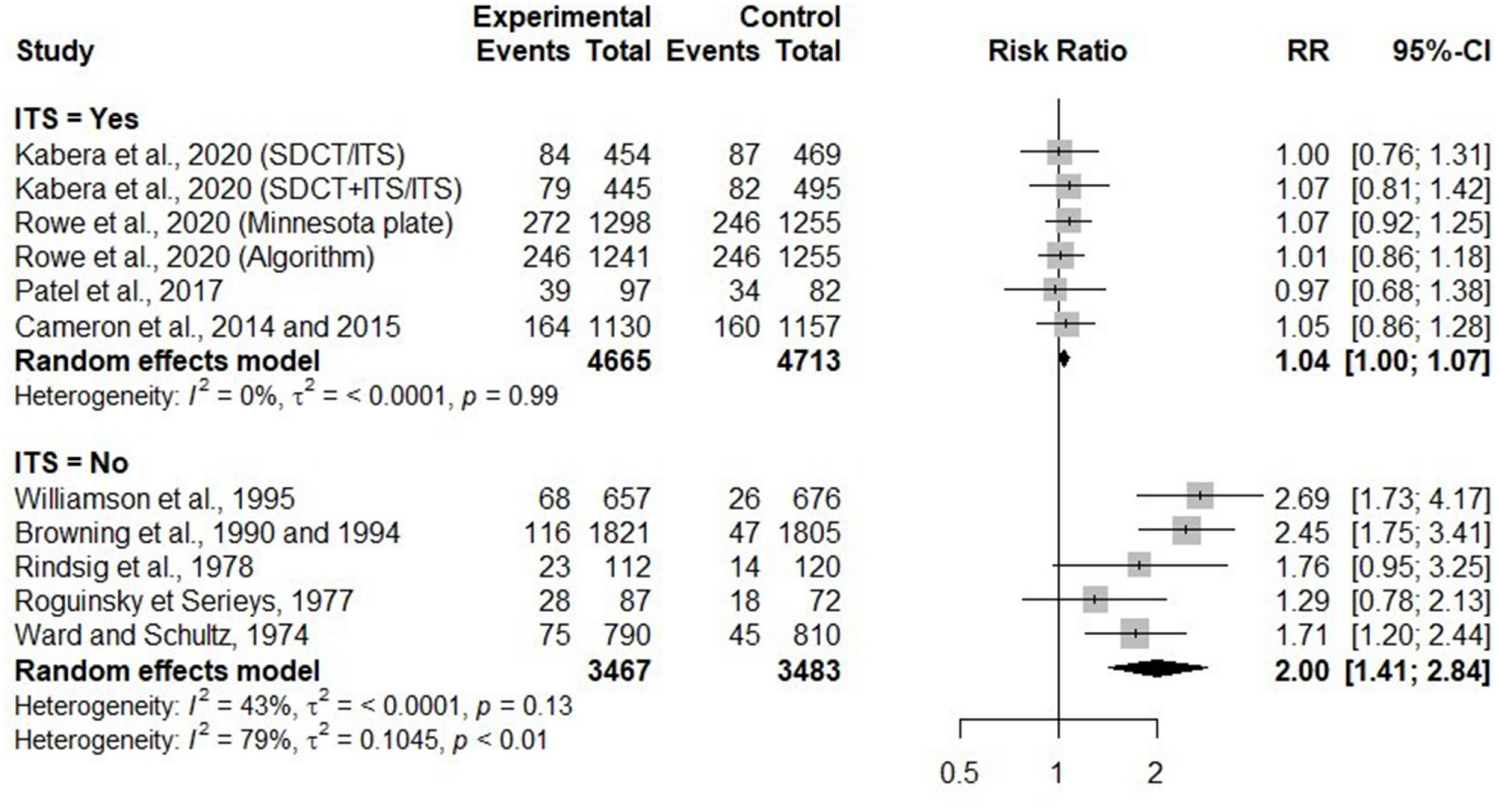
Forest plots showing the effect of selective dry cow treatment compared with blanket dry cow therapy on risk of acquiring new IMI during the dry period, grouped by studies where untreated cows/quarters with antimicrobial received an internal teat sealant (ITS = Yes) and those where they did not receive an internal teat sealant (ITS = No).

In 11 trials, IMI incidence risk during the dry period was investigated and reported at the quarter level. When comparing IMI incidence over the dry period in SDCT and BDCT, one trial characteristic [whether an ITS was applied for untreated (healthy) cows/quarters] was significantly associated with the estimate effect size (*p* < 0.01; tau^2^ = 0.00). An ITS consisting of 65% bismuth subnitrate was used in the six trials where it was applied for untreated (healthy) cows/quarters. Figure 4 presents the RR comparing risk of acquiring a new IMI over the dry period between selective and BDCT for each trial, as well as summaries of RR for trials using ITS or not.

For studies without ITS, the risk of new IMI during the dry period was significantly higher for selectively treated compared with blanket dry treated cows/quarters (RR = 2.00, 95% CI = 1.41, 2.84). Conversely, for studies where an ITS was used to protect healthy cows/quarters, the risk of new IMI during the dry period was not different for selectively treated compared with blanket dry treated cows/quarters (RR = 1.04, 95% CI = 1.00, 1.07).

#### Effects of Selective Dry Cow Therapy on IMI Elimination Over the Dry Period

**Figure 5:**
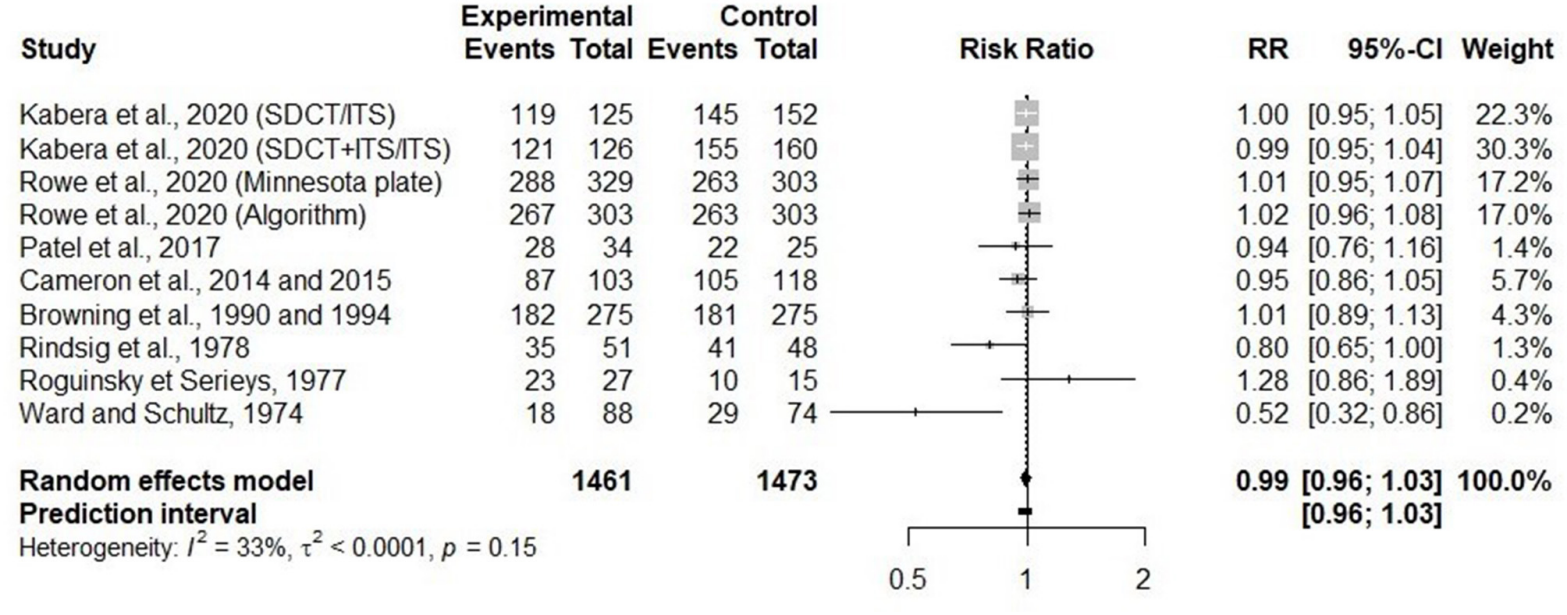
Forest plots showing the effect of selective dry cow treatment compared with blanket dry cow therapy on risk of IMI elimination during the dry period.

In 10 trials, elimination of IMI during the dry period was investigated. None of the variables evaluated in the meta-regressions were significantly associated with the risk of IMI elimination. Figure 5 presents the RR comparing the risk of IMI elimination over the dry period between selective and BDCT for each trial, as well as a summary measure for all trials together. There was no difference (RR = 0.99, 95% CI = 0.96, 1.03) between SDCT and BDCT, regarding the elimination of IMI during the dry period.

#### Effects of Selective Dry Cow Therapy on IMI Prevalence at Calving

**Figure 6:**
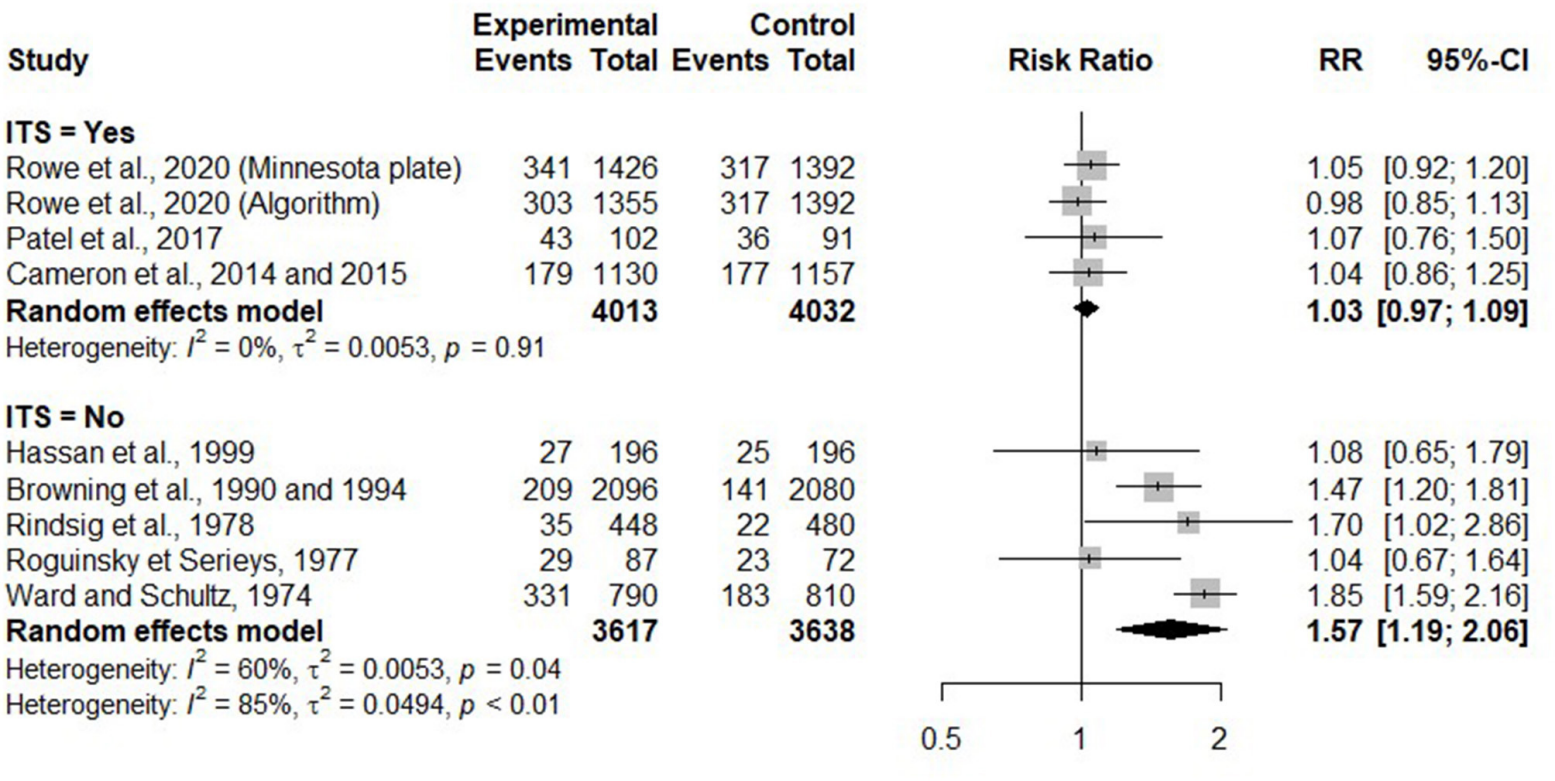
Forest plots showing the effect of selective dry cow treatment compared with blanket dry cow therapy on risk of IMI prevalence at calving.

In nine trials, IMI prevalence at calving was reported. Only one trial characteristic (whether an ITS was applied for healthy cows/quarters) was significant (*p* < 0.01, tau^2^ = 0.01). Figure 6 presents the RR comparing the risk of IMI at calving between SDCT and BDCT for each trial, as well as RR summaries for each category of ITS usage. For trials without ITS (*n* = 5), the risk of IMI at calving was significantly higher for selectively treated cows/quarters than blanket treated cows/quarters (RR = 1.57, 95% CI = 1.19, 2.06), but substantial heterogeneity was still present within this category (*I*^2^ = 60%). For trials using an ITS (*n* = 4), the risk of IMI at calving was not different for selectively and blanket treated cows/quarters (RR = 1.03, 95% CI = 0.97, 1.09). For this latter category, no heterogeneity was seen (*I*^2^ = 0%).

#### Effects of Selective Dry Cow Therapy on Clinical Mastitis Incidence in the Early Lactation

**Figure 7:**
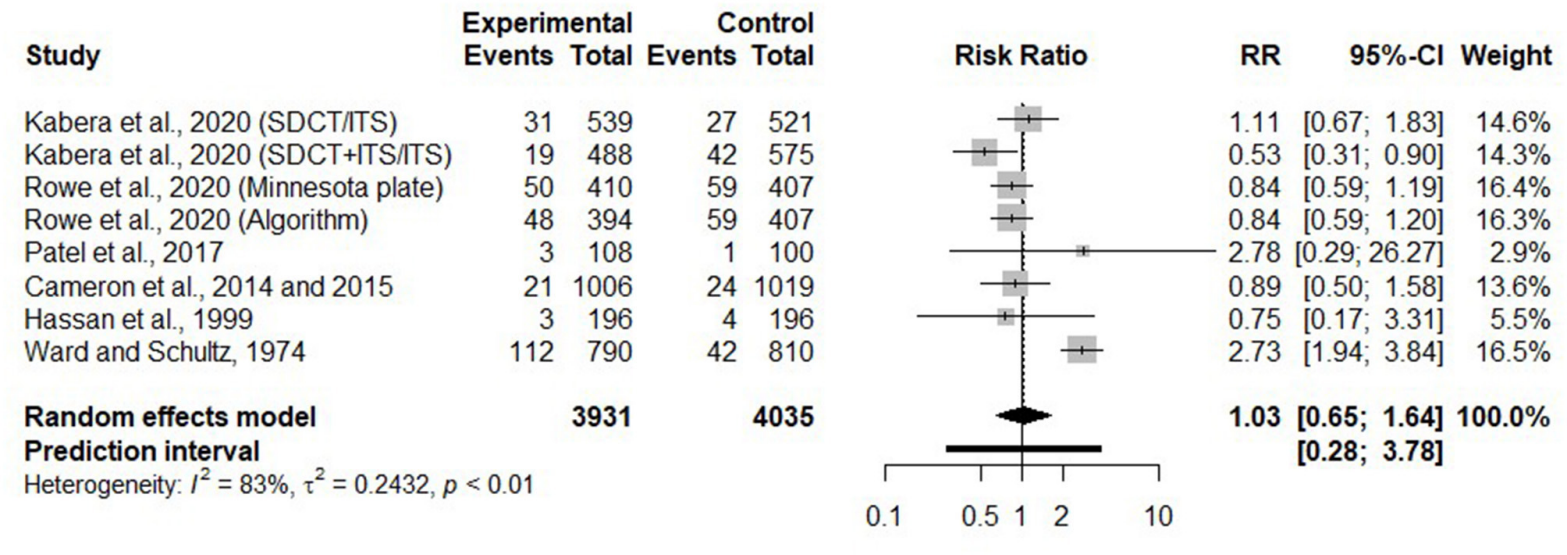
Forest plots showing the effect of selective dry cow treatment compared with blanket dry cow therapy on risk of acquiring CM during the first 4 months of lactation.

Incidence risk of CM early in the following lactation was investigated in eight trials. Two of them reported CM incidence at the cow level and the other six at the quarter level. Before commingling these studies together, it would have been interesting to investigate in a meta-regression the impact of reporting CM, the outcome, at the cow vs. quarter level, but there were not enough trials where CM were reported at the cow level. Among the other potential predictors, only the method used to identify infected cows/quarters at dry off could be tested in a meta-regression and it was not significant. Figure 7 presents the RR of CM incidence during the first 120 days of lactation between SDCT and BDCT for each trial, as well as a summary RR for all trials.

The risk of CM incidence during the first 4 months of lactation was not significantly different between selectively and blanket dry treated cows/quarters (RR = 1.03, 95% CI = 0.65–1.64). However, there was an important heterogeneity among trials (*I*^2^ = 83%). When we considered only the six trials where an ITS was used for healthy cows/quarters, the risk of CM was still not different between SDCT and BDCT (RR = 0.84, 95% CI = 0.65–1.08); however, the heterogeneity was reduced to an almost null value (*I*^2^ = 3%).

#### Effects of Selective Dry Cow Therapy on Milk Yield in the Early Lactation

**Figure 8:**
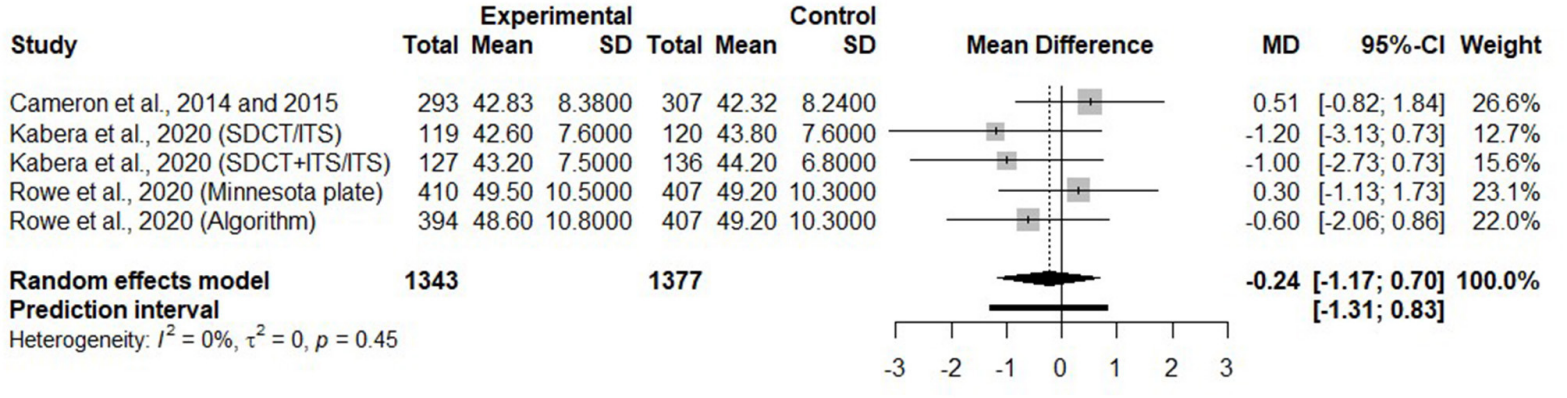
Forest plot illustrating the mean difference in milk production (kg/day) during the first 4 months of lactation after a selective dry cow treatment approach, in comparison with a blanket dry cow therapy.

Only five trials reported on milk yield during the first 4 months of the subsequent lactation. Figure 8 presents the mean difference of milk production during the first 4 months of lactation after a SDCT approach, in comparison with a BDCT, for each trial, as well as a summary measure for all trials. There was no difference in milk yield during the 1st months of the subsequent lactation (MD = −0.24 kg/day, 95% CI = −1.17, 0.70) between SDCT and BDCT.

#### Effects of Selective Dry Cow Therapy on SCC in the Early Lactation

**Figure 9:**
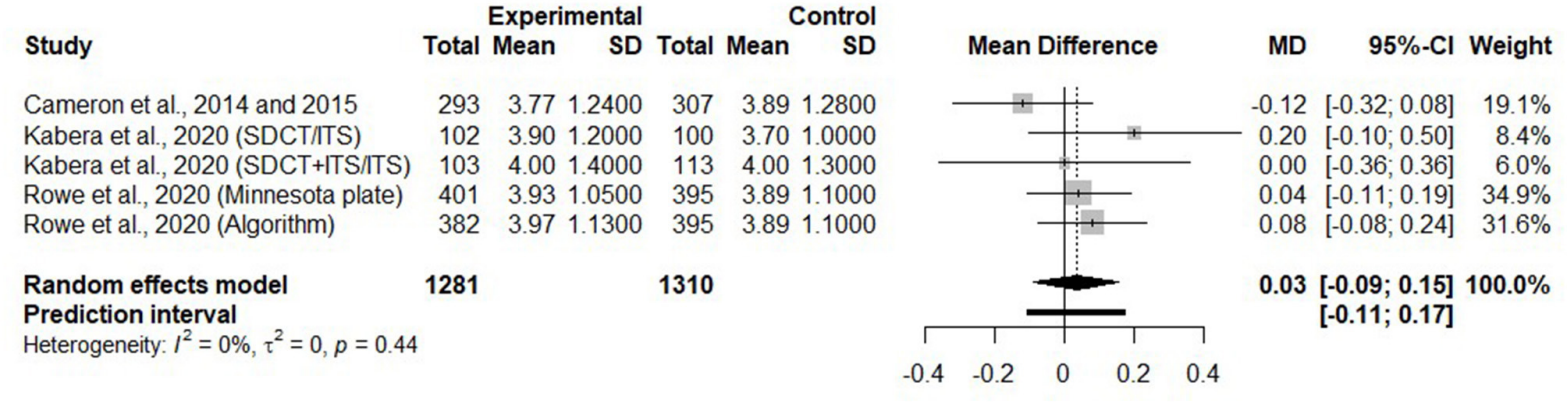
Forest plot illustrating the mean difference in somatic cell counts (on a natural logarithm scale) during the first 4 months of lactation after a selective dry cow treatment approach, in comparison with a blanket dry cow therapy.

Five trials reported on SCC (transformed in ln SCC using the natural logarithm scale) during the first 4 months of the subsequent lactation. Figure 9 presents the mean difference of ln SCC during the 1st months of lactation after a SDCT approach, in comparison with a BDCT, for each trial, as well as a summary measure. There was no difference in ln SCC during the 0–120 DIM period of the subsequent lactation (MD = 0.03, 95% CI = −0.09, 0.15) between SDCT and BDCT.

### Publication Bias

Contour-enhanced funnel plots for each outcome of comparison between SDCT and BDCT are presented in Supplementary Figure 2. However, because of the limited number of available trials, tests for funnel plot asymmetry could not be performed. Therefore, plots were evaluated visually, but it was not possible to identify putative missing studies.

### Summary of Evidence

Table 3 presents a GRADE evidence profile for the different outcomes comparing SDCT and BDCT. Our GRADE assessment indicated a high level of confidence for four of the six studied outcomes/comparisons: (1) risk of acquiring a new IMI in selective dry cow treated quarters/cows when an ITS was administered to healthy quarters; (2) prevalence of IMI, again when an ITS was administered to healthy quarters as part of the selective dry cow protocol; (3) milk yield in the subsequent lactation; and (4) ln SCC in the subsequent lactation.

**Table 3:** GRADE evidence profile: comparison between selective dry cow therapy (SDCT) and blanket dry cow therapy (BDCT) for curing intramammary infections (IMI) at dry off and preventing new IMI during the dry period.

**Outcome and comparison**	**Quality assessment**						**Number of quarters (for IMI) or cows (for CM)**		**Relative risk (95% CI) or Mean difference (95% CI)**	**Quality**
	**# trials (design)**	**Risk of bias**	**Inconsistency**	**Indirectness**	**Imprecision**	**Publication bias**	**BDCT**	**SDCT**		
**IMI incidence**										
ITS to healthy Q/C^a^	6 (RCT)	No serious	No serious	No serious	No serious	No serious	855/4,713	884/4,665	1.04 (0.95, 1.13)	+ + + +High
No ITS to healthy Q/C^b^	2 (RCT) 3 (CT)	Very serious	Serious	No serious	No serious	No serious	150/3,483	310/3,467	1.97 (1.52, 2.54)	+ + – –Low
**Elimination of IMI**	8 (RCT) 2 (CT)	No serious	Serious	No serious	No serious	No serious	1,194/1,455	1,170/1,458	0.99 (0.96, 1.02)	+ + + –Moderate
**IMI at calving**										
ITS to healthy Q/C	4 (RCT)	No serious	No serious	No serious	No serious	No serious	847/4,032	866/4,013	1.02 (0.94, 1.11)	+ + + +High
No ITS to healthy Q/C	3 (RCT) 2 (CT)	Very serious	Serious	No serious	No serious	No serious	394/3,638	631/3,617	1.48 (1.19, 1.84)	+ + – –Low
**CM incidence**	7 (RCT) 1 (CT)	No serious	Very serious	No serious	No serious	No serious	258/4,035	287/3,931	1.03 (0.65, 1.64)	+ + + –Moderate
**Milk yield**	5 (RCT)	No serious	No serious	No serious	No serious	No serious	NA	NA	−0.24 (−1.17, 0.70)^c^	+ + + +High
**ln SCC**	5 (RCT)	No serious	No serious	No serious	No serious	No serious	NA	NA	0.03 (−0.09, 0.15)^d^	+ + + +High

*CM, clinical mastitis; CI, confidence interval; RCT, randomized control trial; CT, control trial (not randomized or randomization not reported); ln SCC, natural logarithm of somatic cell counts*.

a *Where ITS was used for healthy quarters/cows at dry off*.

b *Where ITS was not used for healthy quarters/cows at dry off*.

c *Mean difference in milk yield (kg/day) or in SCC on the natural logarithm scale during the 1st months of the subsequent lactation*.

d *Mean difference in SCC on the natural logarithm scale during the 1st months of the subsequent lactation*.

## Discussion

This systematic review was conducted to determine the efficacy of SDCT (anti-microbial treatment of infected quarters/cows solely) compared with BDCT (all quarters/all cows treated). It reports on SDCT as a potential alternative to BDCT. The main rationale for using a SDCT strategy is to reduce anti-microbial use. This, however, should be achieved, if possible, without any detrimental effect on udder health and milk production. Our results confirm that SDCT can help reduce the use of anti-microbials and that it can be without detrimental effects. However, this was only achieved when IMI incidence in untreated quarters was prevented using an ITS.

A comparable effect of SDCT and BDCT was reported by a review reporting on the prevalence of IMI at calving when all cows received an ITS (12). The same review, in agreement with us, reported a difference between SDCT and BDCT, when an ITS was not used to protect untreated quarters/cows. The importance of the use of ITS at dry off was reported by other previous reviews (8, 29, 30).

The current review also reported on acquisition and elimination of IMI during the dry period and on CM, milk yield, and ln SCC during the subsequent lactation. For all these outcomes, SDCT and BDCT were equivalent, as long as an ITS was used for untreated quarters. However, all trials which reported on milk yield and ln SCC used an ITS. Thus, for those two outcomes, it was not possible to measure the effect of SDCT when an ITS is not used for untreated quarters/cows.

There were small numbers of trials in both ITS categories for all outcomes, but low or no heterogeneity was observed in the ITS category for all tested outcomes (new IMI, prevalence of IMI at calving, and CM during the first 4 months of the subsequent lactation). For trials not using ITS, there was a high risk of new IMI and of IMI at calving in cows/quarters assigned to a SDCT protocol, compared with BDCT, but heterogeneity between trials was still important in this category. This maintenance of heterogeneity may be due not only to a small number of included trials but also to other unmeasured factors which may affect the effect estimated (12).

For all trials, cow- or quarter-level data were used in the meta-analysis, and therefore, clustering of quarters by cow or cows by herd was not accounted for. However, by considering a random effects approach, we accounted for clustering of individuals within different studies.

Regarding the reduction of antimicrobial use in dairy cows at dry off, we conclude that when SDCT is applied, antimicrobial use could be reduced by 66% (95% CI = 49, 80) compared with BDCT. For that outcome, a bimodal distribution was observed, with eight trials reporting proportions in the range of 43–68% and two trials with proportions of 81 and 96%. However, in these trials reporting proportions of 81 (24) and 96% (15), selection of treated quarters was based on a high NAGase (N-acetyl-beta-D-glucosaminidase) value or the occurrence of clinical mastitis during 1 month prior to drying off, respectively.

Moreover, 112 additional antimicrobial infusions during the dry period and early lactation were reported by Ward and Schultz (15). In total, 37 positive quarters including 10 clinical mastitis were reported by Hassan et al. (24) during the dry period in the selective group. These two latter SDCT approaches indeed resulted in very large reduction in antimicrobial usage at dry off, but also in substantial usage of antimicrobials during the dry period.

### Summary of Evidence

#### Impact of Selective Dry Cow Therapy on Preventing the Acquisition of New Intramammary Infections During the Dry Period

Regarding the prevention of IMI over the dry period, we conclude with a high level of confidence that SDCT is as efficient as BDCT when an ITS (65% bismuth subnitrate) is used for untreated healthy quarters/cows at dry off. The efficacy of ITS in the prevention of IMI has been reported in previous reviews (8, 29–31). When an ITS was not used, we would conclude toward a higher risk of new IMI in SDCT compared with BDCT, but with a low level of confidence. These results suggest that, in the countries and through the different time periods where these studies were conducted, the infection pressure during the dry period was too important for leaving quarters completely unprotected (i.e., without antimicrobial and without ITS).

Regarding applying the selection at cow or quarter levels, we did not detect a difference between these SDCT approaches for IMI prevention. However, Halasa et al. (29) reported BDCT to be more protective of new IMI than SDCT when selection was based at the quarter level (RR = 2.01, 95% CI = 1.34, 3.02), but to no significant difference when selection was based at the cow level (RR = 0.52, 95% CI = 0.12, 2.31). In this latter review, however, SDCT protocols of the included studies did not include an ITS for untreated, healthy quarters or cows.

#### Impact of Selective Dry Cow Therapy on the Elimination of Existing Intramammary Infections During the Dry Period

Regarding the elimination of existing IMI present at dry off, we conclude with a moderate level of confidence toward the comparable efficiency of SDCT and BDCT. For that comparison, our level of confidence was mainly affected by the multimodal distribution observed for RR point estimates, with one trial reporting RR estimate of 1.28 (20), one trial with RR estimate of 0.52 (15), and eight trials with RR estimates in the 0.80–1.02 range. However, heterogeneity for this comparison was low (*I*^2^ = 32.8%) and the predicted RR interval was the same as the confidence interval of the effect size from the random effects model (0.96–1.03). A similar efficiency between SDCT and BDCT was also reported by Halasa et al. (32). When Ward and Schultz (15) was omitted, the RR was the same (RR = 0.99, 95% CI = 0.96, 1.03), but no heterogeneity was still seen in the analysis (*I*^2^ = 0).

#### Impact of Selective Dry Cow Therapy on Intramammary Infection Prevalence at Calving

Regarding IMI prevalence at calving, we concluded with a high level of confidence regarding the comparable efficiency of SDCT and BDCT, when an ITS (65% bismuth subnitrate) was used for untreated healthy quarters/cows. The same conclusion was reported by Winder et al. (12).

Conversely, when an ITS was not used, we had a low confidence in our general conclusions. The level of confidence was mainly affected by the bimodal distribution observed for RR point estimates and by a very serious ROB. Almost all trials included in this comparison were older (published between 1974 and 1999), and many of the important information on randomization (e.g., random sequence generation, allocation concealment) were not reported. As it was also reported by Winder et al. (12), when an ITS was not used, there was an increased risk of IMI at calving for SDCT compared with BDCT and a substantial heterogeneity was noted in this subgroup. The presence of a high residual heterogeneity indicates that there is more than one effect within the trials where an ITS was not used. The predicted RR interval within this subgroup was 0.74–2.93.

#### Impact of Selective Dry Cow Therapy on Clinical Mastitis Incidence Early in the Subsequent Lactation

We have a moderate level of confidence regarding the equivalence of SDCT compared with BDCT for the reduction of CM in the following lactation. The level of confidence was affected by a bimodal distribution observed for the estimated RR. However, when we exclude two trials where an ITS was not used for untreated healthy quarters/cows at dry off, the heterogeneity was very low. The importance of ITS in the reduction of CM incidence in the subsequent lactation was reported by previous reviews (8, 30).

#### Impact of Selective Dry Cow Therapy on Milk Yield and ln SCC During the Subsequent Lactation

Concerning milk yield and ln SCC during the subsequent lactation, we conclude with a high level of confidence regarding the comparable efficiency of SDCT and BDCT. However, only trials published between 2014 and 2020 and where ITS was used for untreated healthy quarters/cows at dry off were included in this comparison. None of the previous reviews reported on these two outcomes. In fact, those outcomes were not commonly reported in older studies. However, one of the included trials (7) reported SCC, but on an arithmetic scale which could not be compared with the logarithmic scale. We were not able to reach the authors to get these latter data on a logarithmic scale.

### Comparisons With Published Reviews

The fact that the review of Winder et al. (12) was conducted concurrently to our review provided an opportunity for comparing how our different methodologies affected the presented results. The most striking difference between the reviews are the outcomes analyzed. The main rationale for adopting selective dry cow treatment is the associated reduction in the use of antimicrobials. Quantifying this potential reduction was, in our opinion, essential. Likewise, the risk of CM, milk yield, and SCC in the early next lactation are also important parameters to quantify, to better inform producers considering moving to a selective treatment approach. Finally, although IMI incidence and elimination rates are somewhat captured by measuring IMI prevalence at calving, reporting on these indices provides a better understanding of the underlying biological processes. Our analyses indeed confirmed that the increased IMI prevalence at calving in SDCT protocols when an ITS is not used was mainly caused by an increased IMI incidence in untreated quarters.

Beyond the different outcomes presented, our different methodologies also affected article selection. Three articles included in (12) were not included in our review. The first article (33) was excluded from our review because the antimicrobials used were not specified. Furthermore, it was not clear whether infected cows in the selective group received the same antimicrobial as the cows in the blanket group. When the first author was contacted, he confirmed that each farm used the intramammary antibiotic which was normally used before the trial, but he could not confirm that cows of the same herd and allocated to the selective or blanket groups received the same antimicrobial, as the antimicrobials used could have been modified by a farmer during the study. The second article (34) was excluded, as we considered that cows in the SDCT and BDCT groups were managed differently. In fact, in this latter study, cows in the BDCT group were teat dipped after each milking, while in the SDCT group, they were not teat dipped. Thus, the study of Robinson et al. (34) actually compared blanket dry cow therapy with teat disinfection vs. selective dry cow therapy and no teat disinfection. Moreover, Winder et al. (12) included the Serieys and Roguinsky (35) paper, while our review considered the Roguinsky and Serieys (20) paper. These two papers reported on results of the same trial. The 1977 paper was judged more complete by our team and was, therefore, chosen for inclusion. The results presented in the 1975 and 1977 papers differed slightly and this resulted in (12) using 23/82 quarters with an IMI at calving for blanket treated cows for the Roguinsky's study while our review considered 23/72 infected quarters at calving for blanket treated cows for that same study. Finally, one paper (27) published after the publication of the review of Winder et al. (12) was included in our review and used for comparing prevalence of IMI at calving.

Another difference between our review and that of Winder et al. (12) was observed in the numbers extracted from the study of Cameron et al. (5). In their review, Winder et al. (12) mentioned 164/1,130 and 160/1,157 quarters with a prevalent IMI at calving for the SDCT and BDCT groups, respectively. These numbers were incorrectly extracted in the review of Winder et al. (12). In the paper of Cameron et al. (5), these numbers are indeed presented, but they represented the new IMI risk over the dry period, not the post-calving IMI risk, which were presented in a different table. These numbers were 179/1,130 and 177/1,157 quarters with a prevalent IMI at calving for the SDCT and BDCT groups, respectively.

Overall, these differences in selected studies and in data extraction between reviews resulted in very small differences in the estimated summary measures. Using data from 3,750 quarters, Winder et al. (12) reported a summary risk ratio (95% CI) of 1.09 (0.92, 1.28) when comparing the risk of IMI at calving using selective dry cow therapy with a teat sealant for untreated quarters compared with blanket dry cow therapy. Using data from 8,045 quarters, we reported a risk ratio of 1.03 (0.97, 1.09). On the other hand, we were also able to report on the reduction of antimicrobial usage, IMI incidence risk, and IMI elimination risk, as well as CM incidence, milk yield, and ln SCC in the beginning of the subsequent lactation.

Beyond the review of Winder et al. (12), two other previously published meta-analyses (29, 32) have investigated the comparison of SDCT vs. no dry cow treatment or SDCT vs. BDCT for the prevention of new IMI and elimination of existing IMI during the dry period. In our review, only studies comparing SDCT and BDCT were retained. Thus, only a small number of articles used in the reviews of Halasa et al. (29, 32) are included in this review (7, 22–24). Moreover, none of the studies included in the comparison of SDCT and BDCT (7, 22–24, 36) had used ITS for untreated, healthy quarters or cows.

### Limitations

A small number of trials were included in our review. Those trials were published over a wide period of time (1974–2020). Herd-level inclusion criteria were not reported for six trials. For trials which did, herds were selected with a low BTSCC (<250,000 cells/ml of milk) (5, 6, 25, 27, 28) or a wide range in BTSCC (100,000–400,000 cells/ml of milk) (21, 22). Moreover, Cameron et al. (5, 25) reported on cows with a SCC <200,000 cells/ml on the last three DHI tests and no CM on the same period.

Most reviewed studies (mostly the more recent ones) and, in particular, studies where ITS was used for healthy and untreated quarters were conducted in herds with a relatively low bulk-tank SCC <250,000 cells/ml. For herds with higher bulk milk SCC, there would probably be a higher prevalence of IMI at dry off and especially a higher prevalence of contagious pathogens. Thus, there might be an increased risk of IMI during the dry period for quarters that were not treated at dry off, regardless of receiving an ITS. So, the results of this review should be extrapolated to low SCC herds (BTSCC <250,000 cells/ml) only.

There were also differences in the definition of IMI used across different trials, and the time when the post-calving samples were collected also varied between studies (Supplementary Table 1). These differences in IMI definition could be one of the important causes for the heterogeneity of effect observed between studies.

We initially planned to investigate the effect of randomization (randomized vs. non-randomized trials) in our meta-regressions. However, there was no information on randomization for four studies (20–24). They reported that subjects were allocated randomly, but the description of the randomization process was not detailed. In our descriptive work, these studies were, therefore, classified simply as controlled trials. These studies with no mention of randomization were, however, mostly older studies. Perhaps, at that time, it was not common to mention randomization in the text. Thus, it is unclear whether these studies were truly non-randomized or if the information on randomization was simply lacking in the text. To avoid inappropriate categorization, we did not conduct meta-regression based on reporting or not a randomization.

Meta-regression suggested that the use of teat sealants for quarters/cows not treated with an antimicrobial could explain part of the heterogeneity in the original analysis and reduces the negative impact of SDCT on udder health and milk production in the subsequent lactation. More research would be needed to investigate other factors explaining heterogeneity in the effect estimates.

Another potential limitation was the language restriction, as only articles in English and French were evaluated for inclusion in our review. Thus, we could hypothesize that additional articles would possibly have been included if this restriction was not applied. Also, because of a small number of included trials in each comparison, the potential publication bias could not be thoroughly investigated.

## Conclusion

From the available literature, we can conclude that, for low SCC herds (BTSCC < 250,000 cells/ml), SDCT is as efficient as BDCT for curing existing IMI at dry off, preventing new IMI during the dry period, and preventing CM in the beginning of the subsequent lactation if ITS (65% bismuth subnitrate) is used for healthy, untreated quarters/cows. Moreover, milk yield and ln SCC in the beginning of the subsequent lactation would not differ between quarters treated using a selective or a blanket treatment approach. Finally, we can conclude that the use of SDCT would have an important impact on the use of anti-microbials at dry off in dairy cows.

## Data Availability Statement

The raw data supporting the conclusions of this article will be made available by the authors, without undue reservation.

## Author Contributions

FK co-ordinated the team and was responsible for searching articles from databases and drafting the manuscript. MA was responsible for searching articles from conference proceedings. FK, J-PR, and SD were responsible for extracting data and assessing ROB and their analyses. All authors reviewed and provided feedback on the manuscript, contributed to the development of the review protocol, and selection of eligible articles.

## Conflict of Interest

The authors declare that the research was conducted in the absence of any commercial or financial relationships that could be construed as a potential conflict of interest.

## Abbreviations

SDCT: selective dry cow therapy
BDCT: blanket dry cow therapy
IMI: intramammary infections
CM: clinical mastitis
ITS: internal teat sealants
SCC: somatic cell counts
DIM: days in milk
ROB: risk of bias.
