# Supplementary material for: Comparing Blanket vs. Selective Dry Cow Treatment Approaches for Elimination and Prevention of Intramammary Infections During the Dry Period: A Systematic Review and Meta-Analysis
Source: Front Vet Sci. 2021 Jun 15;8:688450. doi: 10.3389/fvets.2021.688450 (PMC8240810; doi:10.3389/fvets.2021.688450)
Supplement: Supplementary file 1 [file Data_Sheet_1.PDF]

Supplementary Material

1 Supplementary Figures and Tables

1.1 Supplementary Tables

Supplementary Table 1. Follow-up period and definitions of intramammary infection (IMI) at dry off and calving, of new IMI and of elimination of IMI during dry period in 13 articles representing 12 trials included in a systematic review comparing selective dry-cow therapy and blanket dry-cow therapy.

Authors/year	IMI	Eimi[1]	NIMI[2]	Follow up period
Ward & Schultz, 1974	(1) A microorganism was isolated from two consecutive samples or (2) a microorganism must be isolated once and a leucocyte count of the foremilk must be above 1,000,000 cells/ml and/or a CMT score of 2 + or 3 +. Samples were taken within 1 week, about 2 weeks and about 4 weeks after calving	A microorganism that was present at dry off was not isolated from any samples taken after calving	Not reported	CM: 30 DIM<br>Milk yield: Not followed<br>SCC: 30-60 DIM
Roguinsky & Serieys, 1977	Isolation of one or more pathogens in the first monthly milk sample after calving	Absence at calving of pathogen isolated at dry off or isolation of a different pathogen	Isolation of a pathogen which was not present at dry off	CM: Not followed<br>Milk yield: Not followed<br>SCC: Not followed
Rindsig et al., 1978	1) a microorganism was isolated from two consecutive samples taken within 1 week and at 2 weeks post-calving, or 2) a microorganism was isolated once and CMT[3] score ≥ +2 or somatic cell number ≥ 1×10^6 cells/ml.	A microorganism was eliminated if it has not been isolated from any samples taken after calving	1) a microorganism was isolated in both post-calving samples with no microorganism prior to drying off, or 2) the microorganism post-	CM: Not followed<br>Milk yield: Not followed<br>SCC: 1 – 56 DIM

	Corynebacterium bovis was not considered a pathogen and was excluded in determining infections and rates of infection		calving differed from the microorganism prior to drying off	
Browning et al., 1990	Two or three consecutive milk samples contained the same major pathogen (samples taken within 12 h of calving and at the next two consecutive milking)	Not applicable	Infections found in previously uninfected quarters at drying off	CM: 5 months<br>Milk yield: Not followed<br>SCC: Not followed
Browning et al., 1994	Not reported	Not reported	An infection that was identified at calving or during lactation in a quarter that had been uninfected at the previous drying off	CM: 5 months<br>Milk yield: Not followed<br>SCC: Not followed
Williamson et al., 1995	Same organism cultured from both foremilk duplicate samples taken 1–4 days<br>post-calving	Not reported	Not applicable	CM: 8 months<br>Milk yield: Not reported<br>SCC: Not reported
Hassan et al., 1999	Isolation of pathogen on culture of a sample taken at calving. *Corynebacterium bovis* and miscellaneous infections considered to be of minimal importance were excluded	Not applicable	Not applicable	CM: 3 weeks after calving<br>Milk yield: Not followed<br>SCC: Not followed
Cameron et al., 2014	≥100 CFU/mL of milk of any pathogenic organism of interest at either	A pathogen isolated in the dry off sample was considered	A pathogen was cultured at calving on both	CM: 120 DIM.

	of two samples taken at 3–4 and 5–18 DIM. For NAS, a definition of ≥200 CFU/mL was used	eliminated over the dry period if it was absent in both post calving samples (3-4 and 5-18 DIM)	samples (3-4 and 5-18 DIM) and that was not present at dry off	Milk yield: Not followed<br>SCC: Not followed
Cameron et al., 2015	Not applicable	Not applicable	Not applicable	CM: Not followed<br>Milk yield: 180 DIM<br>SCC: 180 DIM
Patel et al., 2017	≥100 CFU/mL of milk of any pathogenic organism of interest, except for NAS and Bacillus spp where spp ≥200 CFU/mL and ≥500 CFU/ml were used, respectively	A pathogen isolated in the dry off sample was considered eliminated over the dry period if it was absent in the post calving sample<br>If a quarter had a mixed infection at dry off (2 pathogens), the absence of both pathogens was required for that quarter to be considered eliminated	The presence of 1 or 2 new pathogens in the post calving sample that were not previously observed in the dry off sample	CM: 30 DIM<br>Milk yield: Not followed<br>SCC: Not followed
Kabera et al., 2020	≥100 CFU/mL[4] of milk of any pathogenic organism of interest	A specific pathogen species found at dry off and absent in the first post-calving sample. If a quarter was infected with two pathogens at drying off, the absence of both pathogens was required at calving	A specific pathogen species not found in the drying off sample and present in the first post calving sample	CM[5]: 1 – 120 DIM<br>Milk yield: 1 – 120 DIM<br>SCC[6]: 1 – 120 DIM
Rowe et al., 2020a	Not applicable	Not applicable	Not applicable	CM: 1 – 120 DIM<br>Milk yield: 1 – 120 DIM<br>SCC: 1 – 120 DIM

Rowe et al., 2020b	≥100 CFU/mL of milk of any pathogenic organism of interest, except for NAS[7] and Bacillus spp where ≥200 CFU/mL and ≥500 CFU/ml were used, respectively	A quarter with a species-level IMI present at enrollment that was not isolated in the post-calving sample	A quarter with a species-level IMI at calving that was not originally present in the enrollment sample	Not applicable

[1]Elimination of intramammary infection; [2]New intramammary infection; [3]California mastitis test; [4]Colony forming units per milliliter of milk; [5]Clinical mastitis; [6]Somatic cell counts; [7]No aureus Staphylococcus.

1.2 Supplementary Figures

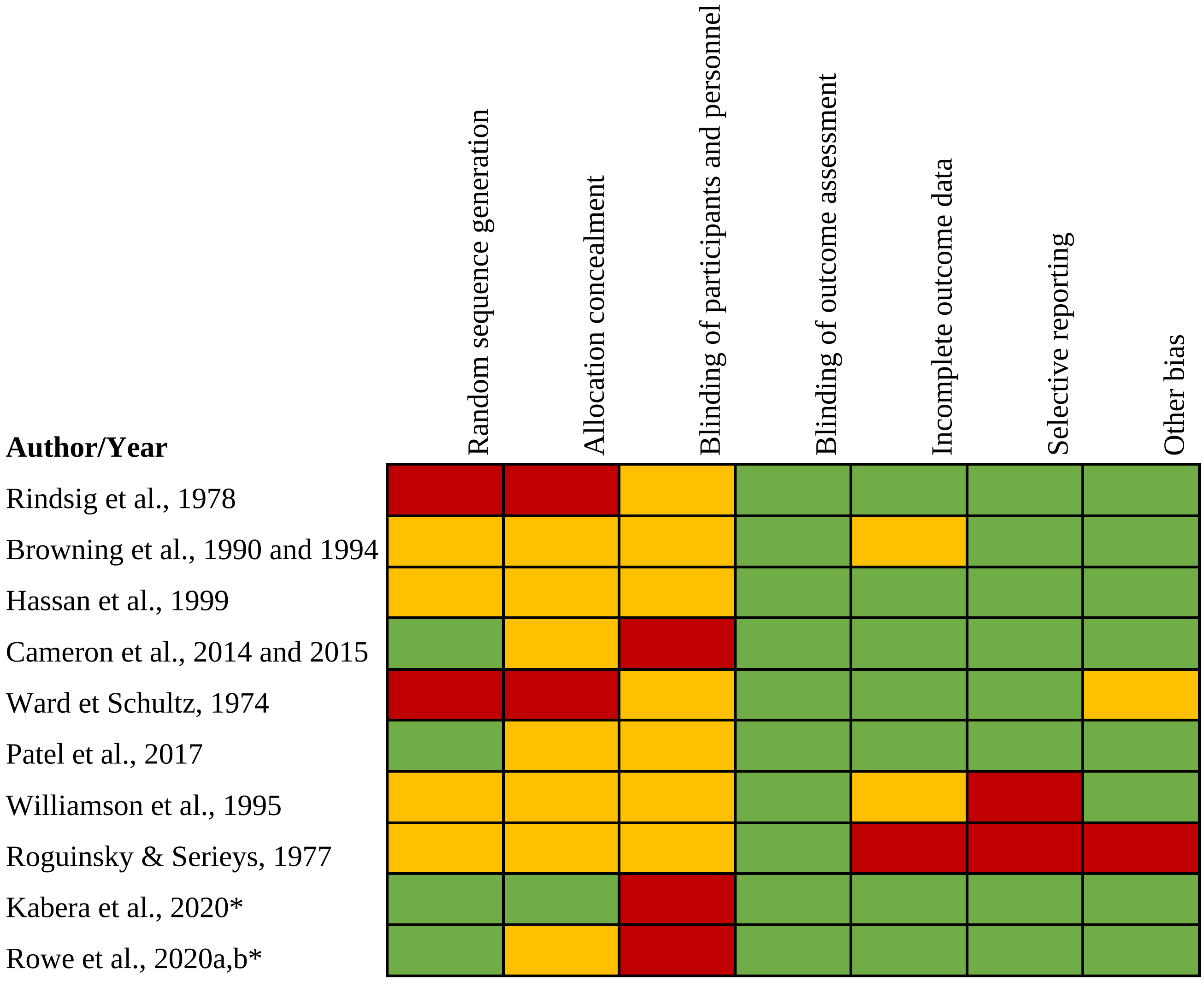

*** Each of these studies reported on two trials**

Supplementary Figure 1. Risk of bias for 12 trials included in a systematic review comparing selective dry-cow therapy and blanket dry-cow therapy for elimination and prevention of intramammary infections.

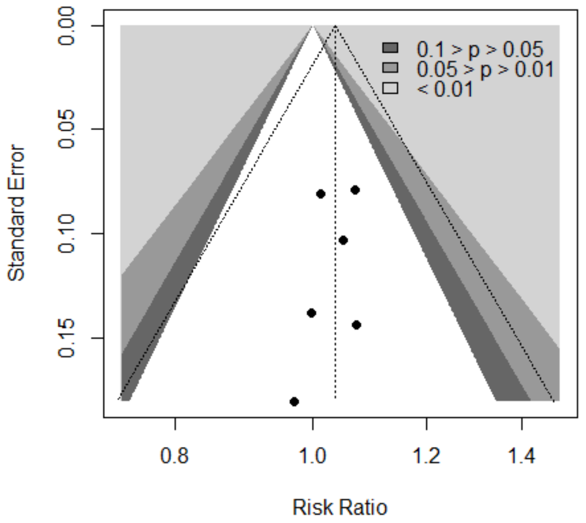

(A) New infections during dry period, ITS

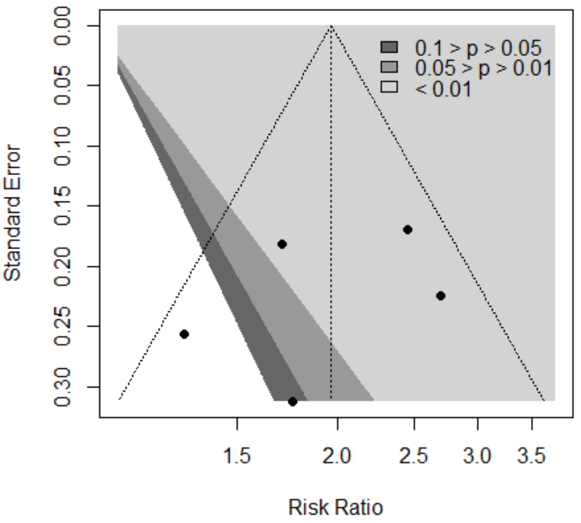

(B) New infections during dry period, no ITS

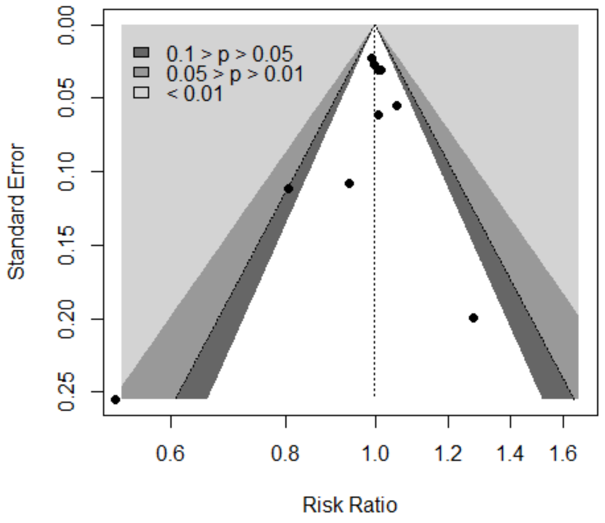

(C) Elimination of IMI during dry period

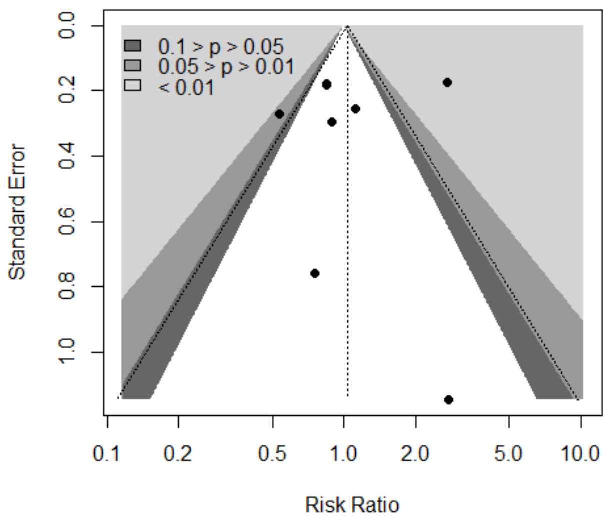

(D) Clinical mastitis

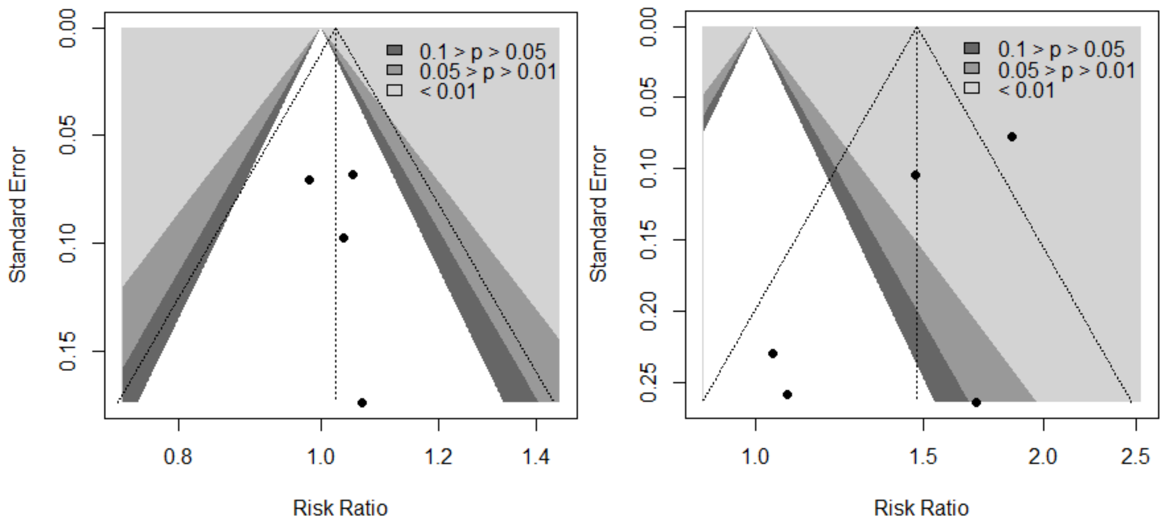

(E) Prevalenve at calving_ITS **(F) Prevalence at calving_noITS**

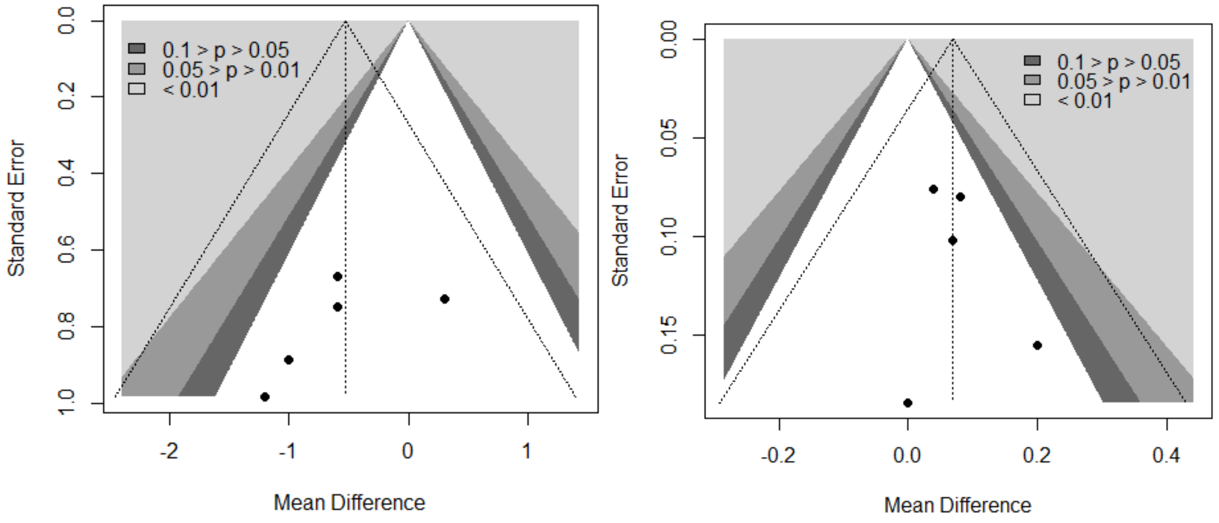

(G) Milk yield **(H) ln SCC**

Supplementary Figure 2. Contour-enhanced funnel plots illustrating potential publication bias for five outcomes investigating the comparison of selective and blanket dry cow therapy: A) IMI incidence during dry period, when an internal teat sealant is used; B) IMI incidence during dry period, when an internal teat sealant is not used; (C) Elimination of IMI during dry period; D) Clinical mastitis incidence during the first days of the subsequent lactation; E) IMI prevalence at calving, when an internal teat sealant is used; F) IMI prevalence at calving, when an internal teat sealant is not used; G) Milk yield during the first days of the subsequent lactation; H) ln SCC during the first days of the subsequent lactation.
